# Diffusion Tensor Imaging Analysis Along the Perivascular Space (DTI-ALPS) in Normal Pressure Hydrocephalus: A Review of Recent Advances

**DOI:** 10.7759/cureus.81830

**Published:** 2025-04-07

**Authors:** Sonali Vij, Carson Brooks, April Pivonka, Zain Abidin, Manisha Koneru

**Affiliations:** 1 Neurosciences, Cooper Medical School of Rowan University, Camden, USA; 2 Neurointerventional Surgery, Cooper University Health Care, Camden, USA

**Keywords:** biomarkers, brain, diffusion tensor imaging, neuroimaging, normal pressure hydrocephalus

## Abstract

Glymphatic dysfunction is linked to neurodegenerative diseases, and imaging markers of this dysfunction may aid in diagnosis and prognosis. Glymphatic dysfunction has been proposed as a key mechanism in the pathogenesis of normal pressure hydrocephalus (NPH). Advanced magnetic resonance techniques, especially diffusion tensor imaging, have been used to evaluate glymphatic function. Diffusion tensor imaging analysis along the perivascular space (DTI-ALPS) is a noninvasive metric that correlates with glymphatic function and has been recently studied in a variety of neurodegenerative diseases. We aim to summarize studies evaluating the association between DTI-ALPS index values and NPH diagnosis and outcomes. Current studies suggest lower DTI-ALPS index values in NPH patients compared to healthy controls. The DTI-ALPS index correlated with other imaging-based markers of NPH and clinical endpoints. However, limitations of the current literature include small cohort sizes; future studies are needed in larger, heterogeneous cohorts to validate these trends. Thus, the DTI-ALPS index shows promise as a valuable tool for diagnosing NPH, predicting treatment response, and assessing disease progression.

## Introduction and background

The glymphatic system is a cerebral waste clearance pathway using astrocyte channels to continuously exchange CSF between perivascular spaces (PVSs) and the interstitium [1]. Fresh CSF flows in from the periarterial spaces, moves interstitial fluid containing metabolic waste products into the perivenous spaces, and then travels to the cervical lymphatic system for removal [2,3]. Dysfunction in glymphatic-mediated elimination of toxic proteins has been implicated in the development of neurodegenerative diseases such as Alzheimer’s disease (AD), Parkinson’s disease (PD), and normal pressure hydrocephalus (NPH) [4-6]. Consequently, imaging-based markers of glymphatic dysfunction may be useful in the diagnosis, treatment guidance, and prognosis of neurodegenerative diseases [7]. Advanced MRI techniques, particularly diffusion tensor imaging (DTI), have been recently applied to visualize and evaluate glymphatic dysfunction. Acquisition of DTI constitutes a brief set of MRI spin sequences additional to that acquired as part of the standard of care for brain imaging and thus is readily compatible with existing imaging workflows. Diffusion tensor imaging analysis along the perivascular space (DTI-ALPS) is a quantitative imaging metric thought to be a noninvasive surrogate for assessing the degree of glymphatic function [8]. There is currently a gap in imaging markers sensitive and specific to NPH, particularly when attempting to distinguish it from other similar, clinically overlapping neurodegenerative diseases. This descriptive review will examine early experience with how DTI-ALPS is capable of characterizing glymphatic function, with an emphasis on clinical applications in the diagnosis and prognosis of NPH.

## Review

Acquiring DTI-ALPS

DTI is an MRI-based technique that quantifies the directionality and magnitude of water molecule diffusion within tissues [9]. DTI imaging required to calculate DTI-ALPS can be performed using standard MRI protocols and increases the overall scan time by no more than ten minutes, highlighting its practicality for assessing glymphatic dysfunction [10]. DTI-ALPS is calculated by the ratio of diffusion in the PVS direction to the diffusion of free water in the interstitium. In order to isolate diffusion in the PVS direction from the diffusion in the surrounding white matter fibers, DTI-ALPS is calculated from the area next to the body of the lateral ventricles, where the major white matter fibers each lie orthogonal to the direction of the PVS. The medullary veins primarily run perpendicular to the lateral ventricle wall (x-axis), while the projection fibers run in the superior-inferior direction (z-axis), and the association fibers run in the rostral-caudal direction (y-axis) [11].

**Figure 1:**
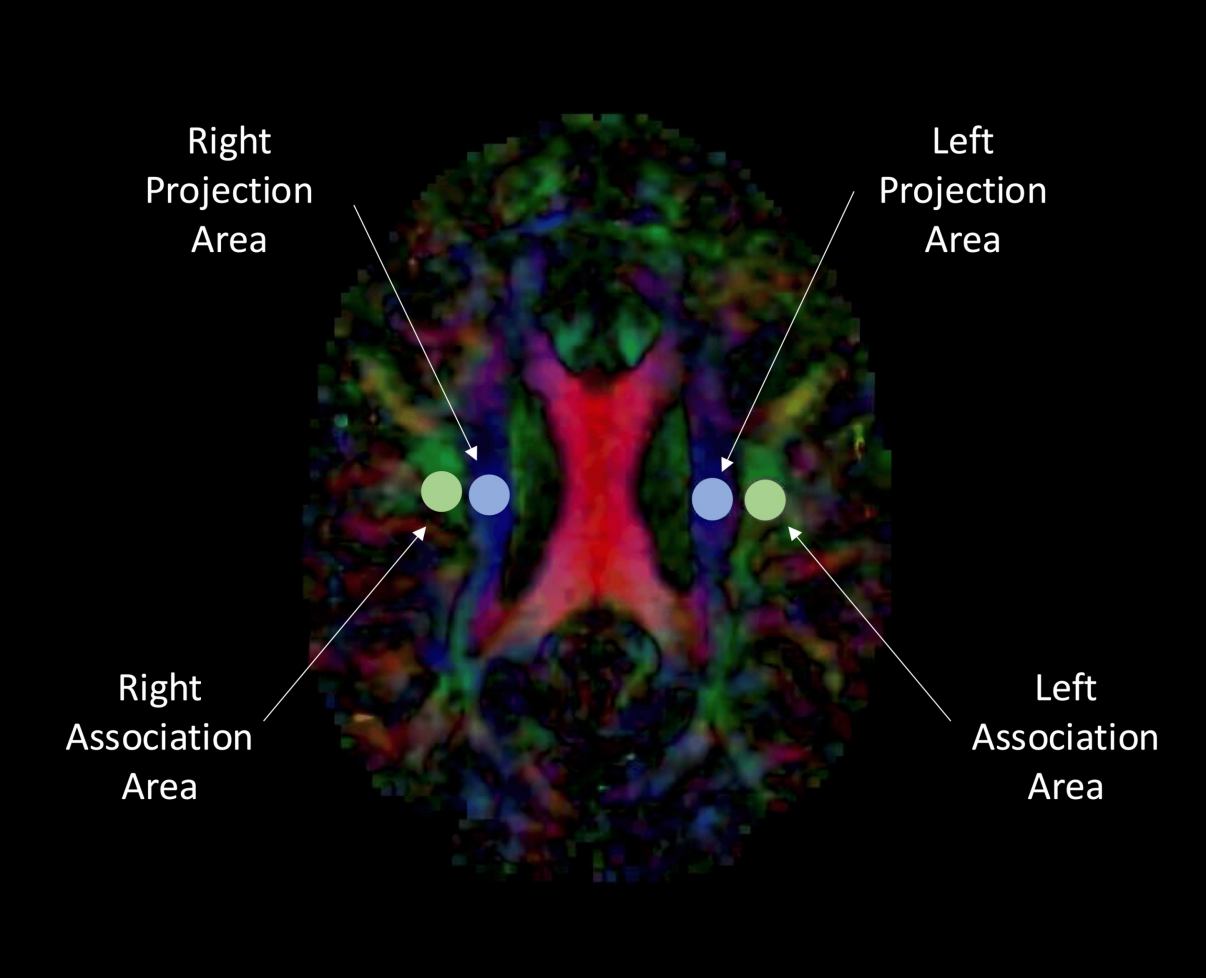
ROI placement for DTI analysis Axial MRI of the head demonstrating diffusion tensor tracks. ROIs are placed in the left and right hemispheres in the projection and association areas for DTI-ALPS index calculation. DTI, diffusion tensor imaging; DTI-ALPS, diffusion tensor imaging analysis along the perivascular space; ROI, region of interest The image was generated using 3D Slicer software, Version 5.8.1 (3D Slicer, Earth, TX, USA).

In an axial brain MRI, two circular regions of interest (ROIs) are placed over the projection fiber region and the association fiber region (Figure 1). Diffusion in all three axes is measured within each ROI. Mathematically, the ALPS index is a ratio between the means of the x-axis diffusivity in the projection area and the x-axis diffusivity in the association area in the numerator and the means of the y-axis diffusivity in the projection area and the z-axis diffusivity in the association area in the denominator [11]. This is calculated in each hemisphere, and the average of left and right ALPS index hemisphere values yields the overall DTI-ALPS index. A higher ALPS index implies a greater magnitude of glymphatic system diffusion in the perivascular direction compared to free water diffusion in other directions [11].

Applications in neurodegeneration

Growing evidence supports the use of DTI-ALPS in evaluating glymphatic dysfunction and clinical correlations in neurodegenerative diseases, such as PD and AD. In a longitudinal study performed by Huang et al. (2024), the ALPS-index scores were found to be lower in AD patients compared to patients who were healthy or with mild cognitive impairment. Furthermore, the study showed the use of the DTI-ALPS index correlated with the extent of disease burden and progression. Lower DTI-ALPS indices correlated with an increased Aβ-PET burden and worsening clinical cognitive decline, as signified by preclinical Alzheimer’s cognitive composite, memory, and executive function composite scores [5].

In PD, impaired glymphatic clearance of α-synuclein and Aβ are theorized to drive disease progression, pointing to a role for DTI-ALPS in assessment [4,12]. A study by Shen et al. (2022) found lower ALPS-index scores in patients with PD compared to controls, with reduction initially confined to the left hemisphere and progressing bilaterally as the disease advanced, suggesting that PD may originate in the left hemisphere [4]. DTI-ALPS may also serve as a marker of disease progression; Ma et al. (2021) noted a statistically significant difference in ALPS-index in patients with late-stage PD compared to early-stage PD and normal controls [13].

Beyond AD and PD, DTI-ALPS has shown promise in detecting glymphatic dysfunction in other neurological disorders, including multiple sclerosis and rapid eye movement sleep behavior disorder (RBD). A retrospective study found lower ALPS-index scores in both relapsing-remitting and progressive multiple sclerosis as compared to healthy individuals, with patients in advanced disease stages showing the greatest reductions [14]. Similarly, in regard to RBD, Taoka et al. (2024) reported a statistically significant lower ALPS index in the RBD patient group as compared to the PD or control group [14]. This finding is noteworthy, as RBD often precedes other α-synucleinopathies, including PD and dementia with Lewy bodies. Follow-up data further demonstrated that higher ALPS-index scores were associated with a reduced likelihood of progression from RBD to these conditions [10]. Overall, DTI-ALPS has demonstrated potential as a useful indicator in assessing glymphatic dysfunction in a variety of neurological diseases.

Applications in NPH

NPH is a neurological disorder characterized by Hakim’s triad of urinary incontinence, gait abnormalities, and cognitive decline. Imaging findings in idiopathic NPH demonstrate ventriculomegaly with no other physical obstruction to CSF flow (i.e., tumor, mass, etc.) [15]. Additionally, NPH may be identified on imaging by other features, including an Evans index >0.3, disproportionately enlarged subarachnoid space, widened Sylvian fissures, and a callosal angle less than 90 degrees. Diagnosis is challenging due to heterogeneous clinical and imaging findings, the significant overlap in symptoms and findings between NPH and other neurodegenerative diseases, as well as the estimate that AD is comorbid with NPH in 18-75% of cases [16]. Moreover, NPH lacks any specific diagnostic features, with 51% of patients not presenting with the classic Hakim’s triad of symptoms [17]. The current mainstay of treatment is CSF diversion procedures such as ventriculoperitoneal shunting, which relieves symptoms by draining excess CSF from the ventricles into the peritoneal cavity [18]. The gold standard for determining treatment response is the CSF tap test, which assesses symptom improvement following the removal of CSF, but its low sensitivity renders it unreliable in excluding patients [19]. CSF diversion has shown favorable outcomes in approximately 74% of patients, as per a meta-analysis conducted by Salih et al. (2024), indicating room for improvement in determining which patients will respond favorably to treatment [20,21].

Although the precise pathogenesis of NPH remains unclear, glymphatic dysfunction has been proposed as a key mechanism. NPH is a communicating hydrocephalus, meaning there is no physical obstruction to CSF flow, but rather inefficient clearance, as demonstrated by Ringstad et al. (2017), who revealed slowed glymphatic clearance through intrathecal administrations of gadobuterol, a CSF tracer [22]. The glymphatic system relies on aquaporin-4 channels located on astrocytic perivascular endfeet, which are found in decreased density in NPH patients [23]. Arterial pulsatility, a critical driver of glymphatic function, is often impaired in NPH patients, further diminishing CSF clearance [22]. Since the glymphatic system is most active during sleep, sleep disturbances are particularly harmful to its function. Obstructive sleep apnea, which is observed in 65-90% of idiopathic NPH patients, exacerbates glymphatic dysfunction through mechanisms such as intracranial venous hypertension and hypoxemia [24]. Given these findings, DTI-ALPS has emerged as a promising diagnostic tool for NPH, capable of detecting glymphatic impairment and providing valuable insights into its overlap with other neurodegenerative disorders.

Recent studies have been performed to elucidate the diagnosis and prognostic value of DTI-ALPS for idiopathic NPH patients. To identify recent studies within the past five years, PubMed was searched for papers published between 2019 and 2024 using the following keywords: “diffusion tensor imaging normal pressure hydrocephalus” or “diffusion tensor imaging along the perivascular space normal pressure hydrocephalus.” Studies were included if the patient cohort included at least one group with primary NPH and excluded if they were case reports or primarily examined other neurodegenerative diseases, pseudo-NPH, or secondary causes of NPH.

The results from the identified studies consistently demonstrate the utility of the DTI-ALPS index in differentiating NPH from healthy controls and other conditions (Table 1). Hasegawa et al. (2024) identified a negative correlation between the ALPS index and CSF medullary pressure, which was highly sensitive to changes, suggesting its potential role in early diagnosis [25]. Lower DTI-ALPS values correlate with markers of disease severity, such as reduced callosal angle and increased Evans index, demonstrating its utility in assessing clinical impairment [26]. Lastly, higher DTI-ALPS values are associated with positive tap test responses and improved Timed Up and Go test performance [6,25].

**Table 1:** Summary of literature assessing the DTI-ALPS index in NPH patients between 2019 and 2024 * Pseudo-NPH was defined as suspected NPH but was determined to be an alternative diagnosis due to neurological examination and/or failure to respond to the tap test. DTI-ALPS, diffusion tensor imaging analysis along the perivascular space; NPH, normal pressure hydrocephalus; P-NPH, pseudo-normal pressure hydrocephalus; TUG, Time Up and Go test

Study	Number of participants	ALPS index trend (NPH vs. other groups)	ALPS index and imaging markers	ALPS index and treatment outcomes
Bae et al. (2021) [6]	NPH: 16	↓	(-) correlation with Evans index	N/A
	Control: 16			
Hasegawa et al. (2024) [25]	NPH: 30	N/A	N/A	(-) correlation with the TUG index
Georgiopoulos et al. (2024) [26]	NPH: 30	↓	N/A	N/A
	P-NPH*: 27			
Yokota et al. (2019) [27]	NPH: 12	↓	(+) correlation with callosal angle	↑ post-test in tap test responders
	P-NPH*: 12			
	Control: 12			

Although early studies have demonstrated promise for DTI-ALPS in NPH, there are limitations to this technique. Ventricular enlargement may confound the interpretation of DTI-ALPS findings in NPH by causing white matter deformities. These deformities risk disrupting the placement of the ROIs, which must be accurately positioned for proper orthogonal alignment of the medullary vessels with respect to projection and association fibers. When this alignment is lost, the ALPS index may capture diffusion from unrelated fibers, resulting in misleading conclusions about glymphatic dysfunction [14]. The challenge of proper ROI placement is further compounded by the fact that this process is manual, thereby increasing the risk of error [28]. It is important to note that while ventriculomegaly affects certain white matter tracts, such as the cingulum bundle and thalamic radiation, Wu et al. (2024) found that most white matter changes seem unrelated to ventricular size, pointing to other pathological processes at work [28]. Consequently, proper ROI placement to yield representative DTI-ALPS values necessitates careful, patient-specific evaluation of anatomical features that may affect glymphatic assessment accuracy. Understanding these processes is crucial to properly using DTI-ALPS to assess metrics such as clinical severity since certain white matter changes are tied to key NPH symptoms like gait disturbances and cognitive impairment. Improving our understanding of the factors that affect the ALPS index is key in guiding clinical decisions, developing more accurate tools and processes to address these challenges, and optimizing patient outcomes.

Future directions

The DTI-ALPS method represents a promising noninvasive tool for assessing glymphatic function, but further validation against established measures of glymphatic activity, such as intrathecal contrast administration and dynamic contrast-enhanced MRI, is essential in better understanding its reliability as a surrogate for glymphatic activity [29,30]. Such validation is critical for ensuring that DTI-ALPS can reliably detect glymphatic dysfunction across different patient populations and clinical scenarios, including differentiating between NPH and other clinically similar neurodegenerative diseases. Moreover, accessibility to DTI may be limited in clinical settings that are unable to perform DTI-specific sequences; consequently, future research may elucidate approaches using a diverse range of existing systems, including synthetic post-processing methods, to facilitate widespread clinical adoption. Additionally, the development and adoption of standardized protocols for DTI-ALPS acquisition and analysis, particularly adequate ROI placement in patients with challenging anatomical features, are vital to improving reproducibility. Protocol standardization will reduce variability in data collection, interpretation, and post-processing protocols, allowing for more meaningful comparisons across studies and facilitating the integration of DTI-ALPS into broader research frameworks [31].

Another area of exploration is the use of DTI-ALPS in longitudinal studies monitoring glymphatic function to track disease progression and evaluate treatment responses in conditions like NPH. This is particularly relevant for emerging treatments such as the CereVasc e-Shunt (CereVasc Inc., Charlestown, MA, USA), which is a novel, endovascularly placed shunt for idiopathic NPH patients currently being evaluated as part of an active clinical trial (NCT05232838). Potentially incorporating DTI-ALPS as a biomarker in clinical trials could provide valuable data on how interventions influence glymphatic dynamics, potentially opening new avenues for therapeutic monitoring and disease progression [32].

Furthermore, combining DTI-ALPS with other imaging modalities and biomarkers may elucidate relationships between glymphatic clearance, metabolite deposition, and other pathophysiological processes contributing to disease progression [33-36]. These integrated approaches may improve our understanding of the mechanisms driving neurodegenerative disease progression, thus elucidating additional potential therapeutic targets.

## Conclusions

Recent advances have been made in the use of DTI-ALPS in NPH to assess glymphatic function. With ALPS-index scores being a marker for diagnosis, disease severity, and treatment outcomes, DTI-ALPS offers a promising method for distinguishing NPH from overlapping neurological conditions, thus allowing for better-informed patient care. Future research should focus on validating DTI-ALPS and standardizing protocols to enable its integration into routine clinical practice.
